# Society Meetings

**Published:** 1901-08

**Authors:** 


### SOCIETY MEETINGS.

---

The American Association of Obstetricians and Gynecologists will hold its fourteenth annual meeting at the Hotel Hollenden, Cleveland, O., Tuesday, Wednesday and Thursday, September

17, 18 and 19, 1901, under the presidency of Dr. William E. B. Davis, of Birmingham, Ala. The committee of arrangements is composed of Dr. M. Rosenwasser, 722 Woodland Avenue and Dr. William H. Humiston, 122 Euclid Avenue, Cleveland, either of whom may be addressed concerning rooms or other local information regarding the meeting.

The following list of papers is offered:

1. The president's address, William E. B. Davis, Birmingham.

2. Indications for the combined vagino-abdominal operation of hysterectomy, Rufus B. Hall, Cincinnati.

3. Title to be announced, Henry D. Ingraham, Buffalo.

4. A method for suspension of the uterus, Robert T. Morris, New York.

5. Tubercular peritonitis, experimental and clinical, John B. Murphy, Chicago.

6. Report of a case of acute pancreatitis and fat necrosis, Edward J. Ill, Newark.

7. Pelvic and abdominal tumors complicating pregnancy, with report of cases, Rufus B. Hall, Cincinnati.

8. Pathology and treatment of hourglass stomach, with report of two cases, Charles G. Cumston, Boston.

9. Early operations in appendicitis and method, Joseph Price, Philadelphia.

10. Title to be announced, J. Henry Carstens, Detroit.

11. The undeveloped uterus, C. L. Bonifield, Cincinnati.

12. An interesting case of tubo-abdominal pregnancy, Wm. H. Humiston, Cleveland.

13. Title to be announced, Walter B. Dorsett, St. Louis.

14. Title to be announced, C. C. Frederick, Buffalo.

15. Report of a case of ruptured tubal pregnancy, Webb J. Kelly, Galion, O.

16. Diseases and injuries of the cervix uteri and their treatment, Joel W. Hyde, Brooklyn.

17. Title to be announced, L. H. Dunning, Indianapolis.

18. Title to be announced, James F. W. Ross, Toronto.

19. Extrauterine pregnancy; report of cases with specimens, George S. Peck, Youngstown.

20. Is Cesarean section justifiable in placenta previa? E. Gustav Zinke, Cincinnati.

21. Title to be announced, Edwin Walker, Evansville.

22. Some reflections on ectopic gestation, David Tod Gilliam, Columbus.

23. Some forms of disease involving the uterine appendages, Augustus P. Clarke, Cambridge.

24. The mechanical, or combined plastic and mechanical treatment of retrodeviations of the womb, M. Rosenwasser, Cleveland.

25. Title to be announced, H. E. Hayd, Buffalo.

26. New method of opening the abdomen in gynecological surgery, Charles G. Cumston, Boston.

27. A second contribution to the surgery of gastric ulcer, Henry Howitt, Guelph, Ont.

---

THE American Electro-therapeutic Association will hold its eleventh annual meeting at Buffalo, Tuesday, Wednesday and Thursday, September 24, 25 and 26, 1901, under the presidency of Dr. Ernest Wende, of Buffalo. The meetings will be held at the Armory of the 74th Regiment, and the headquarters of the association will be established at the Niagara Hotel, where rates for rooms are $2.00 and upwards a day for one person, and members are advised to secure their rooms without delay. Dr. Henry R. Hopkins, 433 Franklin Street, Buffalo, is chairman of the committee of arrangements, and he should be addressed concerning information desired with reference to the meeting.

---

THE Homeopathic Medical Society of the State of New York will hold its semiannual meeting at Buffalo during the last week in September, 1901. To give all in attendance ample opportunity to attend the meetings and yet see the exposition, it has been arranged to hold three half-day sessions, from 9 a.m. to 1 p.m., thus allowing the afternoon and evening for recreation and sightseeing. The place of meeting will be one of the hotels adjacent to the exposition grounds.

---

THE National Association of Homeopathic Members of Examining Boards held its eleventh annual meeting at Richfield Springs in conjunction with the meeting of the American Institute of Homeopathy. Papers and reports were presented by fifteen state examining boards, the subjects outlined therein for consideration covering a wide range of thought bearing on the practical working of state control of medical licensure.

The officers elected for the ensuing year are as follows: President, A. Korndoerfer, Philadelphia; secretary, H. M. Paine, Atlanta; treasurer, E. Cranch, Erie. These officers and S. H.

Calderwood, of Boston, and V. H. Hallman, of Hot Springs, Ark., constitute the executive committee.

---

The Medical Society of the Missouri Valley will hold its fourteenth annual meeting at St. Joseph, on Thursday, September 19, and at Eureka Springs, Friday and Saturday, September 20 and 21, 1901. V. L. Treynor, of Council Bluffs, Iowa, is president, and Charles Wood Fassett, of St. Joseph, Mo., is secretary.

---

All members desiring to contribute to the program of the Buffalo Academy of Medicine for the session 1901-1902 are requested to communicate with the officers of sections, as announced below: Section of medicine—chairman, Julius Ullman; secretary, A. E. Woehnert, 155 Allen St. Section of surgery—chairman, H. R. Gaylord; secretary, H. C. Rooth, 350 Ashland Ave. Section of obstetrics—chairman, Thos. F. Dwyer; secretary, W. Mansperger, 1003 Main St. Section of pathology—chairman, Grover Wende; secretary, Irving P. Lyon, 531 Franklin St. Section of ophthalmology, otology, etc.—chairman, Elmer G. Starr; secretary, Arthur G. Bennett, 26 Allen St.

The council would appreciate the assistance of members in securing the services of out-of-town men of note in the profession to participate in the program. Chas. G. Stockton, President; F. W. McGuire, 1540 Main street, secretary.

The Chautauqua County Medical Society held its annual meeting at Chautauqua, N. Y., in the Hotel Athenæum, July 9, 1901. In the morning, at 11 o'clock, was held the business session at which the following officers were elected for the ensuing year: President, E. A. Scofield, of Bemus Point; vice-president, N. E. Beardsley, of Dunkirk; secretary, C. A. Ellis, of Sherman. Dr. W. S. Bainbridge was first elected president, but declined the honor.

In the afternoon at 2 o'clock a scientific session was held and the following program was observed: The president's annual address, E. A. Scofield; Skin cancers, E. S. Rich; Locomotor ataxia, H. F. Hunt; Tubercular osteitis, McDonald Moore; Cardiac and vaso-motor sedatives, C. W. Southward; The Chautauqua County Medical Society, a retrospect, N. G. Richmond. After the regular program, interesting cases were reported and discussed.

THE American Public Health Association will hold its twenty-ninth annual meeting Monday to Friday, September 16, 17, 18, 19 and 20, 1901, at the armory of the 74th Regiment. The headquarters of the association will be at the Niagara Hotel.

Dr. Henry R. Hopkins, chairman of the committee of arrangements, announces that this place will make for the comfort of those in attendance and the efficiency of the deliberations of the association, that the location is easy to reach, overlooking the waters of Erie and Niagara, well shaded, cool and free from the noises and distractions of the center of the city.

The committee is informed that transportation to and from Buffalo, from all parts of America is already offered at unusually desirable rates, and, further, that still lower fares may be secured before the time of meeting. Rates at the Niagara for rooms are two dollars and upward a day for one person, and members are advised to secure their rooms without delay. Those who desire accommodations elsewhere are requested to write the Visitors' Information Company, 538 Ellicott Square, or the Buffalo Business Men's Association, 217 Main Street. Desirable and convenient accommodations in close proximity to headquarters may thereby be secured at rates for rooms of one dollar and upward a day for each person.

---

THE Medical Association of Central New York will hold its 34th annual meeting at Buffalo, August 27 and 28, 1901. The officers announce that the present plan is to have the first session on Tuesday morning, the 27th, in the lecture room of the Society of Natural Sciences, at the Library Building, corner of Washington Street and Broadway. After luncheon the afternoon can be devoted to clinical demonstrations or operations at several of the hospitals, which members can visit according to their interest in different special departments, or, if they prefer, a preliminary inspection may be made of the exposition. In the evening there will be an informal social gathering, if possible, somewhere on the exposition grounds.

On Wednesday morning the second session will be held at the Library Building, and, after luncheon, the afternoon and evening may be spent at the exposition. It is desired that the papers be limited to ten minutes, and each speaker in the discussions to five minutes.

Another circular letter will be issued later giving the list of papers and further arrangements then completed. The attendance will probably be large and it is hoped that the sessions will also prove specially instructive and enjoyable. Lucien Howe, President, Buffalo.; Charles A. Van der Beek, secretary, 16 Gibbs Street, Rochester.

---

The annual meeting of the Lake Keuka Medical and Surgical Association will be held at Grove Springs, Lake Keuka, N.Y., August 13 and 14, 1901, commencing at 11.30 a.m.

The officers are as follows: President, Henry Flood, Elmira; vice-president, Wm. B. Macy, Willard; secretary and treasurer, W. W. Smith, Avoca.

Program—President's address, Henry Flood, Elmira; discussion opened by Wm. Austin Macy, Willard. Affections of the nervous system in early and late syphilis, George Conderman, Hornellsville; discussion opened by W. Sylvester Cobb, Corning. Cerebro-spinal meningitis, Floyd S. Crego, Buffalo; discussion opened by Robert P. Bush, Horseheads. Treatment of melancholia, Charles G. Wagner, Binghamton; discussion opened by G. D. Crandal, Blossburg. Treatment of lobar pneumonia, Charles G. Stockton, Buffalo; discussion opened by C. L. Stiles, Owego. Relation of mosquitoes to malaria, Arthur W. Booth, Elmira; discussion opened by Veranus A. Moore, Ithaca. The small-pox problem, its dissemination, control and attending difficulties, Ernest Wende, Buffalo; discussion opened by H. D. Wey, Elmira. The necessity of proper diet for infants at the beginning, Charles W. M. Brown, Elmira; discussion opened by Charles W. Hayt, Corning. Tetanus—report of three cases, W. B. Clapper, Farmington; discussion opened by M. L. Bennett, Watkins. Appendicitis, when to operate, A. L. Beahan, Canandaigua; discussion opened by C. S. Parkhill, Hornellsville. The indications and limitations of surgical intervention in diseases of the stomach, W. G. Macdonald, Albany; discussion opened by Lewis W. Rose, Rochester. Surgery of the biliary passages, John B. Deaver, Philadelphia; discussion opened by Roswell Park, Buffalo. Asthma in its relation to nasal disease, John O. Roe, Rochester; discussion opened by G. M. Case, Elmira. Concerning purulent ophthalmia, Lucien Howe, Buffalo; discussion opened by Sherman Voorhees, Elmira. Cancer of the uterus, H. P. Jack, Canisteo; discussion opened by F. W. Ross, Elmira. Early prophylaxis in obstetrics, William A. Howe, Phelps; discussion opened by C. C. Harvey, Dundee. Pathology in the German University, A. T. Kunkel,

Westfield. Remarks on leprosy, J. E. Walker, Hornellsville; discussion opened by H. R. Ainsworth, Addison.

Smoker given by the Physicians of Elmira, N.Y., Tuesday evening, at the Lake Keuka Club.

